# BIM-Based E-Procurement: An Innovative Approach to Construction E-Procurement

**DOI:** 10.1155/2015/905390

**Published:** 2015-05-18

**Authors:** António Aguiar Costa, António Grilo

**Affiliations:** ^1^ICIST, Instituto Superior Técnico, Universidade de Lisboa, 1049-001 Lisboa, Portugal; ^2^UNIDEMI, Faculdade de Ciencias e Tecnologia, Universidade Nova de Lisboa, 2829-516 Caparica, Portugal

## Abstract

This paper presents an innovative approach to e-procurement in construction, which uses building information models (BIM) to support the construction procurement process. The result is an integrated and electronic instrument connected to a rich knowledge base capable of advanced operations and able to strengthen transaction relationships and collaboration throughout the supply chain. The BIM-based e-procurement prototype has been developed using distinct existing electronic solutions and an IFC server and was tested in a pilot case study, which supported further discussions of the results of the research.

## 1. Introduction

In the last few years, as an answer to the increasing need to reduce waste and improve performance, several innovative technologies emerged in the construction sector. New information and communication technologies (ICT) have challenged traditional working methods and stimulated change and modernization, especially in areas of e-business and building information modelling (BIM) [1–8]. Slowly but progressively, these technologies are being integrated into construction processes, demonstrating the prospect of potential gains.

E-business platforms, which may take different forms [9, 10], play an important role as communication and business process management instruments, emerging as an effective support to collaboration, information management, and sharing [11, 12]. The extranets supported by these electronic platforms capture the supply chain communication practices and provide controlled communication, and the relevance of information sharing instruments is becoming progressively evident for the industry [13]. The automated business processes supported by these platforms allow for increasing process efficiency and project management capabilities and leverage the role of information during projects' lifecycles, creating an information-based environment that improves BIM potential and stimulates its implementation. Subsequently, the implementation of BIM emphasizes knowledge sharing throughout the life cycle of a building, making supply chain and life cycle integration possible and improving information management capabilities [14].

Considering these emerging issues, we propose an innovative approach to e-procurement in construction that uses BIM to support e-procurement processes. The hypothesis behind the proposed framework is that BIM-based solutions may reduce the negative effects of the fragmentation of the construction project lifecycle through the integration and integrity of information across the procurement processes in a project's life-cycle. This will imply new strategic approaches to the procurement cycle and support more accurate decisions. The result is an integrated instrument connected to a rich knowledge base capable of advanced operations and able to strengthen transaction relationships and collaboration throughout the supply chain.

In order to achieve this purpose, the paper starts by reviewing the literature regarding e-procurement and BIM, focusing on major developments and most significant impacts on the construction industry. The paper then presents a BIM-based e-procurement model and respective prototype, which was developed to test the validity of the formulated model. Finally, the paper discusses the application of the prototype to a pilot case study, reporting the most important results of the research.

## 2. E-Procurement and Building Information Modelling in the AEC Sector

### 2.1. Developments in E-Procurement in the AEC Sector

According to several authors [15, 16] the use of e-procurement platforms results in a reduction of more than 3% of public expenditures without reduction in outputs. This is possible mostly because e-procurement helps to reduce complexity, improves competitiveness and transparency, and creates an integrated electronic environment to support advanced electronic instruments to manage, and monitor contracts [17].

E-procurement is best viewed broadly as an end-to-end solution, which integrates and streamlines many procurement processes throughout an organization [18]. Some of these procurement processes are the following:

- ex-ante e-evaluation: refers to multicriteria evaluation of needs and procurement strategies;
- e-noticing: concerning electronic publication of public procurement notices;
- e-submission: concerning electronic submission of proposals;
- e-decision: concerning electronic evaluation of proposals, subsequent communication of evaluation results, and discussion and analysis of results;
- e-award: concerning electronic contract awards to suppliers with the best proposals;
- e-ordering: concerning all activities, including sending an order document from public buyers to suppliers, to the transmission of delivery instructions for ordered goods and services;
- e-invoicing: concerning claim for payment for goods and services ordered and delivered under agreed-upon conditions;
- e-payment: agreed electronic payment management and execution;
- e-contract management: refers to the use of electronic contract management instruments to monitor and improve contract performance and document management;
- ex-post e-evaluation: agreed multicriteria evaluation of the contract execution, and the eventual generation of KPIs to support future tendering processes.

In a fully integrated and paperless context, all these processes should be combined and all relevant information must be available electronically. This will allow for a reduction of administrative work and automate operational processes, offering more time to think strategically [19]. On the other hand, it will accelerate market information efficiency, allowing further fine-tuning of procurement decisions such as supplier and proposal evaluations, procurement methods, and negotiation strategies [20].

Considering its advantages, several countries are encouraging the implementation of e-procurement in the public sector [21]. For instance, in Portugal the Public Contracts Code (PCC) was approved by Decree-Law 18/2008 on January 29, 2008, and is in force since July 29, 2008, mandates public e-procurement in Portugal. Currently, public procurement is completely paperless and, gradually, the private sector is recognizing its advantages and implementing e-procurement, especially in the construction sector [17]. This situation raises interesting opportunities for e-procurement service providers and offers an important incentive to construction since it pushes firms and public authorities to modernize and digitalize, obligating the implementation of more responsive, collaborative, and intelligent working systems. Inevitably, the competitive environment around e-procurement service providers stimulates the development of more advanced e-procurement solutions, which integrate not only the entire e-procurement cycle but also the project lifecycles and provide support for innovative procurement and working models [22].

Against the technological potential of the existing electronic instruments, strategic perspectives on procurement and more integrated approaches to supply chains gain momentum and the integration of e-procurement with other industry-oriented ICT, such as BIM, becomes extremely important. The present research reflects this progressively paperless environment and effectively integrates these relevant technologies that are changing the construction paradigm.

### 2.2. Building Information Modelling

BIM has been developed in the last decade with the advent of refined computer aided design (CAD) systems able to enrich virtual 3D models of buildings with complementary data (as physical characteristics, unit costs, fabrication details, etc.). As a new methodology, BIM promotes a more cooperative work between all specialties during the different stages of the construction project and also used during the life cycle of the building allowing a more efficient use of resources, decrease of errors due to lack of information/communication, and a more efficient management of the building operations costs [6, 23, 24].

Increasing use of BIM has been seen in the last few years [25, 26]. This is mostly due to the potential of this technology to improve construction project performance becoming more widely recognized. Eastman et al. [6] argue that BIM technology makes it possible to construct an accurate 3D virtual and parametric model of a building containing precise geometry and relevant data needed to support construction, fabrication, and procurement activities necessary for the building process, which effectively contributes to increase collaboration and project quality. Thorpe et al. [27] also emphasize that by making virtual reality simulations possible, BIM fosters project understanding and emphasizes integrated and coordinated decision-making in supply chains, providing the construction industry with an instrument to support more rigorous and consistent decisions throughout the building's life cycle. It is worth mentioning that several case studies demonstrating effective performance improvements can already be found in the literature [28–31].

Several authors point out that the true benefits of BIM are obtained when the technology is applied throughout the project lifecycle, from design to demolition [32]. However, this leads to a major challenge: the data exchange between all the parties demands seamless interoperability [24, 33]. Efforts to address this problem have been undertaken, mainly related to the development of standard formats, such as the industry foundation classes (IFC) [34, 35]. IFC is an object-oriented interoperable format for representing building product model data and may be the most used format for interoperability purposes.

The construction industry is gradually starting to understand the importance of interoperability in order to achieve integrated and automated work processes, with savings in cost and time [36]. As more disciplines of the construction industry adopt building information modeling, integrated design, and delivery work processes, the need for interoperable applications grows clearer [37]. Lipman et al. observe that industry leaders and government agencies now recognize the importance of addressing the interoperability problem, and numerous reports document the imperative to solve this problem. The National Research Council report [38] on the competitiveness and efficiency of US construction, for example, clearly states that the lack of interoperability is a major source of construction industry waste and inefficiency.

In an interoperable scenario, BIM is a powerful tool to support lifecycle integration [39] and in the particular case described in this paper, to support the e-procurement process. The information contained in the BIM-based design model can be shared directly in an e-procurement process, which can reduce the heavy human workload and manual errors in traditional work. Due to the complexity of construction, e-procurement processes are still time-consuming and prone to error [3].

Having all the information centralized in a specific model may increase process efficiency and accurateness. As Ma et al. [36] mention, highlighting the case of cost estimation, several BIM-based software applications have emerged, such as Innovaya [9] and Vico Estimator [10], in which the BIM-based design model can be imported to perform effective tendering of building projects cost estimation, confirming that the use of BIM throughout the procurement process may realize considerable gains.

In a paperless, interoperable, and information-based environment such as the one encouraged by the use of BIM, interesting synergies can be generated by combining BIM with other ICT, such as e-business platforms. This combination results in hybrid electronic platforms capable of several advanced operations. Asite [40] is an example of these hybrid platforms. In this case, a document and model management instrument are connected to a BIM server, improving information management capabilities. Onuma [41] should also be cited as another type of hybrid platform. In this case, it is a web-based planning system supported by a model based platform essentially oriented to the conception and design construction phase. Although these cases demonstrate that interesting results can be achieved with this hybrid approach, few solutions exist, especially using open formats, such as IFC, and few efforts have yet been made to explore some specific functions, such as the application of BIM for e-procurement. This specific application may be achieved in the near future, however, as BIM is becoming mandatory for public works in several countries, and this specific application may help to leverage construction procurement performance, particularly by emphasizing the role of information throughout the procurement cycle and, mostly, by allowing the automation of several procurement processes, diminishing the probability of errors and processes duration.

## 3. Research Method

The present study was developed as an action research [42] conducted within a business environment, presupposing strong commitment between team members [43]. The research team included members from the following:

- two universities, mainly responsible for the coordination and development of the scientific work;
- an e-procurement service provider that provided the e-procurement platform and respective web services;
- a software house specialized in project management software applications, which provided support on project management features;
- a network of construction partners who gave important feedback on the project and participated in the simulation test.

All participants gave rich, full insights and participated proactively. This participative and collaborative process were based on cycles, each of which comprised several steps: planning, action, and fact-finding about the result of the action. These cycles converged on better understanding the problems and phenomena throughout a learning process based on continuous refinement of methods, data, and interpretation [44]. In the case of the research reported here, the research cycles focused on three major phases:

- exploratory study and proposition of a BIM-based e-procurement framework;
- development of a prototype of the solution proposed;
- simulation and test of the prototype developed using a pilot case study, an experiment with a fixed approach, and a controllable environment.

In the following sections, the most important developments and outcomes achieved during the three major phases of the research are presented, with special attention to the critical issues faced.

## 4. BIM-Based E-Procurement Framework

Joining developments in ICT and procurement in the AEC sector, the authors consider that a BIM-based e-procurement solution not only makes more advanced and intelligent approaches to e-procurement systems possible but also promotes the generation of large electronic and interoperable networks that interact dynamically. The rich information-based environment that is thus created not only strengthens the automation of many operational tasks but also enhances information and knowledge management. This supports more accurate decisions and strategic approaches to the procurement cycle and potentiates more effective collaborative planning, collaborative design, integrated decision, scenario analysis, product comparison, document automation, processes automation, contract management, and performance management. This shifts from traditional e-procurement approaches, where CAD files are exchanged but are mainly used by each agent to print and recreate their own information models, not reusing or integrating information and not contributing to reduce construction projects fragmentation, recognized as a major hindrance to increase construction productivity. With BIM-based e-procurement, information may indeed flow in a more seamless way across application of the various agents within procurement processes.

The proposed solution allows any user to initiate an e-tendering process using a BIM model. For instance, if a contractor wishes to purchase a specific product for a building, he can select that product (or related element) in the BIM model and launch the tendering process using the electronic platform. Automatically, all the necessary information is obtained from the model and the tendering is initiated.

**Figure 1:**
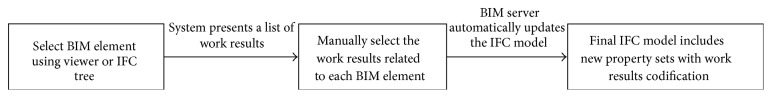
Method to include in the BIM model additional information (such as work results).

It is important to note that the use of BIM for procurement purposes demands a very detailed model, including all necessary information to launch the tendering process, which is a major challenge. Every BIM element must include several information sets such as work results related to each element or product identification (or types of product) in order to allow for the implementation of automatic procurement procedures. The association of this information to BIM elements can be made using the method presented in Figure 1, which can be implemented by an electronic tool that presents a list of organized information related to relevant types of information (work results, products, or other) after the user selects a specific BIM element.

**Figure 2:**
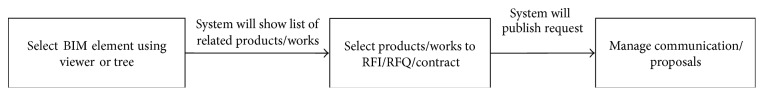
Method to initiate procurement process using BIM model.

**Figure 3:**
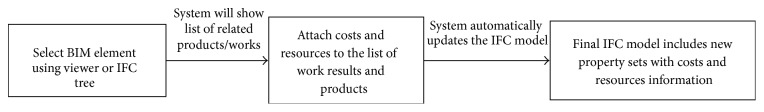
Method to make a proposal using the BIM model.

Although the links must be created manually using taxonomies available, information should be automatically included in the IFC model. To automate this operation the electronic platform should be connected to a BIM server, which should allow creating a new property set in the IFC file in which it includes the information previously associated with BIM elements. It is possible thereafter to initiate the e-procurement process in a simple way (Figure 2). Selecting the BIM element, it is also possible to send “requests for information” or “requests for quotes,” which will guarantee that all information (including messages) are linked to the model. Tenderers may also submit tenders using the BIM model. In this sense they must attach specific information about the products, the costs, and the resources to the model. The process is similar to the one presented previously (Figure 3).

It is important to understand that some information is not easily included in the model. This makes it imperative that the system be able to attach files to elements of the BIM model. In this sense the system should create connections between messages and/or files and the elements of the BIM model. By selecting a determined BIM element (using the viewer of the IFC tree view, which is an hierarchic view of IFC elements), it should be possible to attach and access all the information related to it, not only information within the model but also external information connected to the model previously (messages, files, external links, etc.) [5]. This feature would allow procurers and tenderers to attach information to the model information that is not included in the BIM model.

## 5. BIM-Based E-Procurement Prototype

### 5.1. Use Cases and Front-End Development

The first step toward prototype development was to model traditional workflow for the construction e-procurement lifecycle, which included identifying the various players and respective value-added activities, information flows mapping, and determining major deliverables and decision points.

**Figure 4:**
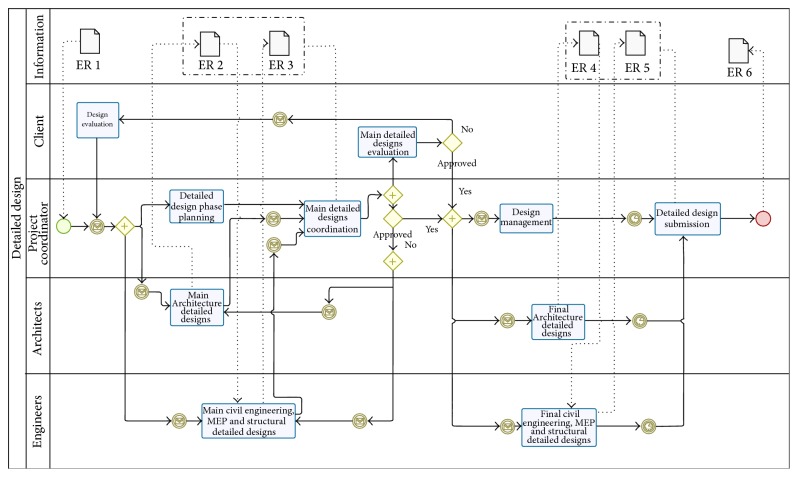
BMPN map example.

Providing a standard visualization mechanism for business processes defined in an execution-optimized business processing language [45], business process model and notation (BPMN) was used to model all traditional processes inherent to construction lifecycle phases (Figure 4) [46]. Special attention was paid to actors, major decisions taken, and information flows; the principal deliverables generated in each phase were also identified and described. Afterwards, BPMN diagrams were analyzed in detail and several modifications were introduced into the workflow to optimize information flows and enhance collaborative work, recurring to existing technologies such as BIM. Existing e-procurement platforms were the starting point for the developments proposed.

The resulting BPMN diagrams supported the development of a functional matrix in which the functionalities of the platform for each phase and each type of user were identified that considered information requirements identified previously. Considering the functional matrix and the BPMN diagrams, the use cases were then defined and, in accordance to use case specifications, front-ends were constructed. Microsoft SharePoint was the instrument used to implement them based on a form metaphor structure consisting of a series of forms (pages) with which the user interacts. Each form contained a number of fields that display output from lower layers and collect user input.

### 5.2. Platform Architecture

To support the BIM-base procurement framework previously described, the electronic platform (called PLAGE platform) was developed integrating and combining four different preexisting solutions. Microsoft SharePoint 2007 was used as the business collaboration platform system and as the front-end and to implement a set of workflow and rule-based procedures for the e-procurement. The EDM Model Server from Jotne EPM Technology was used to implement BIM-based features such as storing and manipulating IFC models. The IFC Engine Viewer provided by TNO was used as an IFC 3D viewer. Vortal eGOV is an e-procurement platform for the AEC sector. The IFC version considered was the IFC2x3 version.

The disparate electronic instruments work seamlessly in an integrated way through Web-services connections. The platform generic technology architecture was grounded on the combination of the latest architectures like model-driven architecture (MDA), the service-oriented architecture (SOA), cloud computing, and building information model (BIM)—the SOA4BIM Framework [47–49]. The application of the SOA4BIM Framework in the context of e-procurement is expected to overcome many technological barriers by reusing much of the standardization and research work done in the BIM and AEC sector, namely, the IFC and STEP standards, and at the same time use current technology, like Web-services, for implementation. To structure the development of the platform, the service-oriented approach was organized into four layers [50]:

- *presentation layer*: providing the application user interface and involving forms for smart client interaction and ASP.NET technologies for browser-based interaction;
- *application services layer*: implementing the business functionality of the application, comprising a number of components implemented using one or more NET programming languages;
- *Business/interoperability services layer*: supporting business services connected with external services using SOAP;
- *data layer*: providing access to external systems such as databases.

### 5.3. IFC Server Implementation

**Figure 5:**
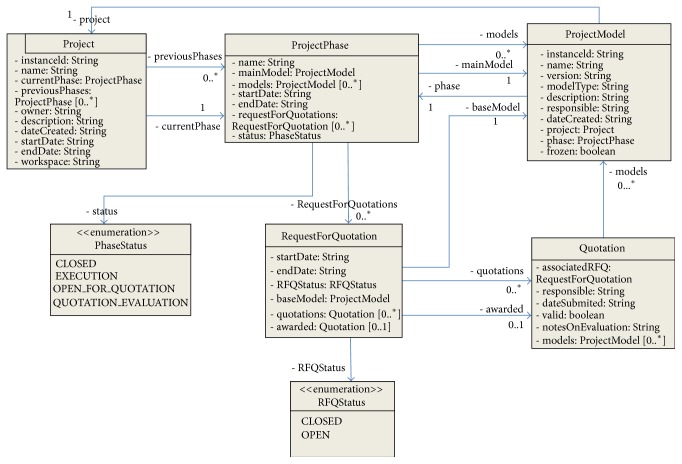
Information model implemented on the EDM server.

**Figure 6:**
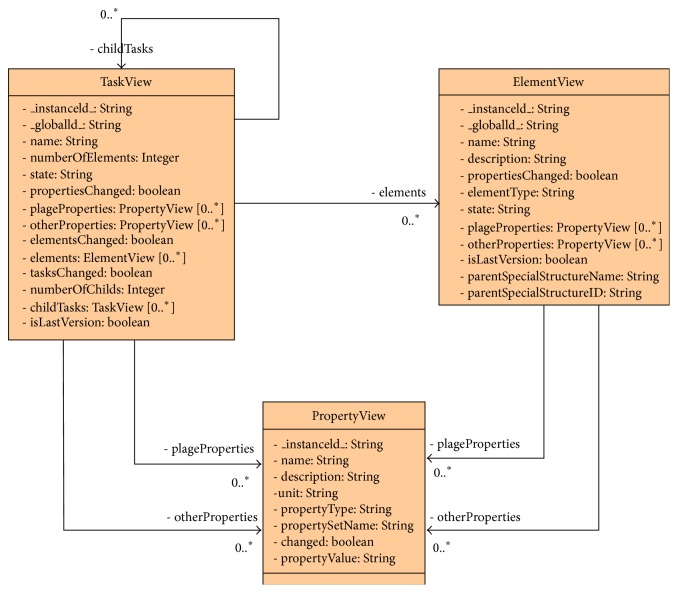
Information model to support client-server information flow.

In the present case the EDM server component was used to perform IFC operations and the client application has been fully developed using Sharepoint framework. Considering the specific requirements of the research project, new information models have been designed and implemented on the EDM server in order to allow an efficient management of the project data (Figure 5) and define the information flow based on the IFC objects included in the IFC model (Figure 6). In this case, the entities are not persistent, and are used only to support information flow between the client and the server. For more information on these information models please see the appendix.

In order to link the BIM elements to the tasks, a connection between the EDM server and the software Primavera CCOP has been implemented using the web services. This allows the EDM server to send information to Primavera CCOP, which generates the list of elements based on the Uniformat standard and supports the manual linking of these elements to the respective work results.

### 5.4. BIM-Based Interface

**Figure 7:**
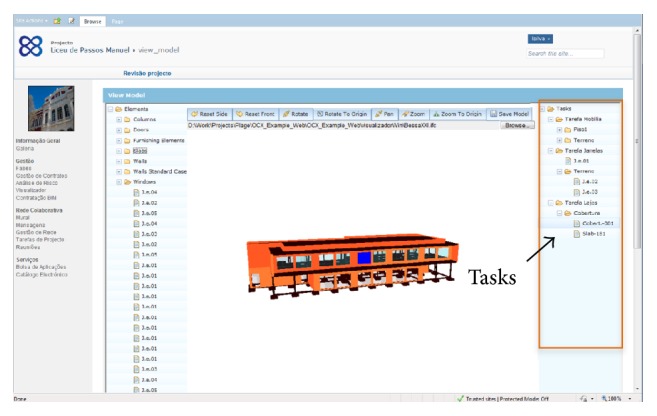
BIM viewer (IFC-based).

The BIM-based interface was created according to the proposed solutions and supports two major advanced features:

- viewing and manipulating IFC models, providing access to all the information contained in the models (Figure 7);
- managing tasks and other information related to each BIM element, from which it is possible to initiate the e-procurement process using the connection implemented with the Vortal e-procurement platform.

As mentioned above, the IFC engine viewer used (provided by TNO) is an IFC viewer with an advanced content mapper based on internal queries, which allows visualizing and interacting with 3D models. It has been embedded in the platform as an applet and integrated in the platform information flows.

## 6. Pilot Case Study: Liceu Passos Manuel

The PLAGE platform prototype was tested using a pilot case study based on a public sector project focusing on the renovation and expansion of a public secondary school, the Liceu Passos Manuel. The public entity responsible for the work provided all necessary documentation, including detailed design, specifications, and contract, and interacted with the research team to explain the most important features of project design and construction. Several other entities, such as major contractors and designers, also provided useful insights to enrich the research study. Throughout the pilot case several issues were addressed regarding the implementation of the innovative BIM-based e-procurement solution developed.

**Figure 8:**
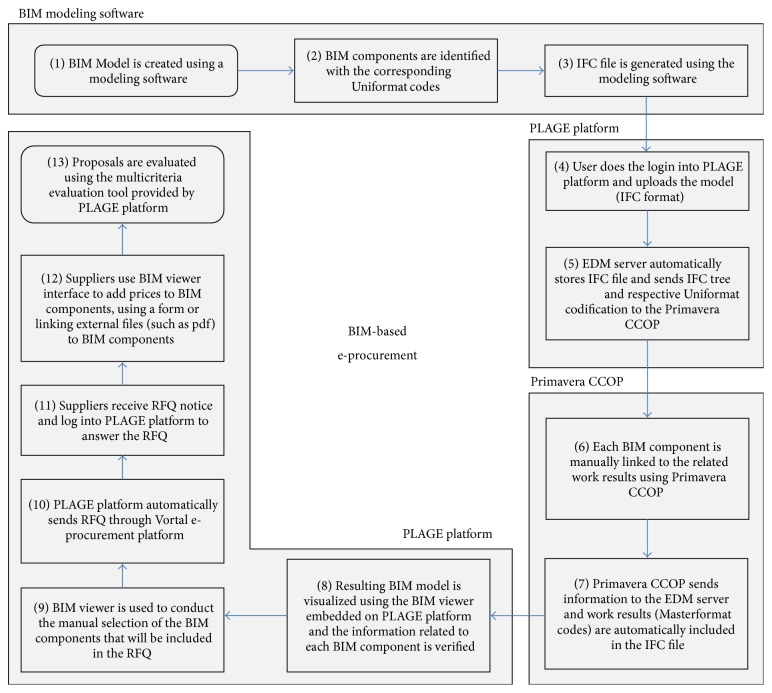
Simulation scenario.

To test the prototype, the design and a part of the procurement process inherent to the Liceu Passos Manuel school project were simulated. The pilot case was structured in 13 major steps, shown in Figure 8.

Throughout these steps the BIM-based e-procurement prototype was tested in various ways, including the following:

- viewing and manipulating BIM models created throughout the pilot case and accessing inherent information;
- using BIM model viewer to attach external files to the model;
- launching RFQ and RFI (requests for information) using BIM-based e-procurement interface;
- initiating e-procurement process based on the elements of the BIM model;
- submitting a proposal using the BIM-based interface.

A brief description of each step is presented below in order to clarify the entire pilot testing process.

**Figure 9:**
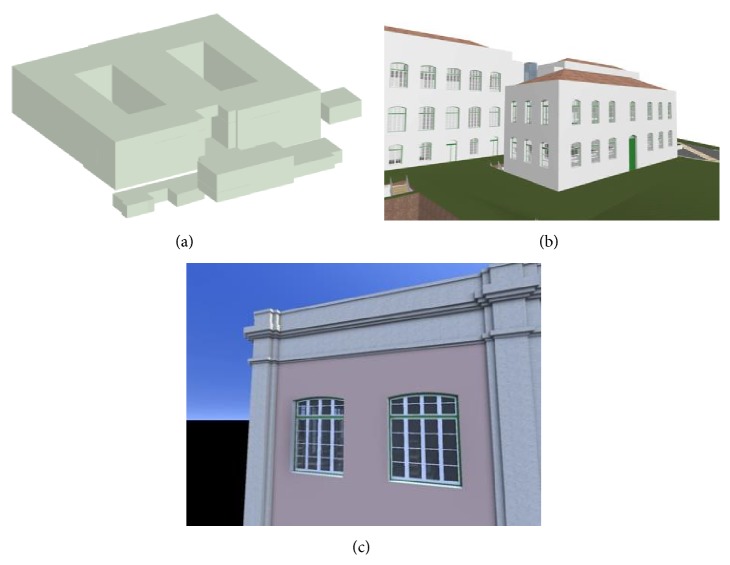
BIM models respecting different levels of detail (LOD100, LOD200, and LOD300, from (a) to (c)).

*Step 1.* In this initial stage several BIM models have been created (the designs provided were in 2D), which were used to test consistency of the BIM-based interface and PLAGE BIM management module. The modeling process reflects four modeling steps with increasing design detail and follows the BIM directives adopted in other countries (Figure 9), such as the US where the* American Institute of Architects* (AIA) identifies several levels of detail for models that correspond to different project phases [51]. Beyond being a BIM-based e-procurement platform, PLAGE is a project workflow and document management application and is thus used to manage models and processes right from the earliest stages, that is, the early program and concept design. The models were created using Archicad software and the design process was delivered in an integrated way, stimulating maximum collaboration between intervening actors. 

*Step 2.* This step focused on the enrichment of the BIM model with information and Uniformat codes, which were manually included in each BIM component. For success, this process requires careful modeling (e.g., avoiding overlap between components of the model) to guarantee that the model is close to reality. The development and use of a BIM objects' library already including all the needed information is recommended.

*Step 3.* The IFC models were automatically generated from the BIM models originally created in the Archicad proprietary format. IFC interoperability concerns were given special consideration during this transformation. Particular attention was given to the problems that may arise due to the lack of interoperability between proprietary formats and IFC formats. As stated by Lipman et al. (2011), user expectations on IFC interoperability are not being met by CAD applications, which have been granted IFC certification and should be able to exchange 100% of the information in their CAD models via IFCs 100% of the time; however, they argue that the use of more adequate and systematized conformance and interoperability testing methodologies may lead to good results in terms of IFC interoperability. Meanwhile, in order to prevent major problems in IFC transformation operations, a careful analysis following IFC file generation should be conducted to assess possible errors. Special attention should be given to ground, materials, tailor-made objects (e.g., windows, doors, etc.), and specific elements subject to any advanced operation conducted by other BIM software (e.g., joints in complex walls or solid construction operations). Despite interoperability issues, IFC is crucial to collaborative work to facilitate a multisoftware approach to projects and to drive performance of construction supply chains to higher levels of interoperability [52, 53].

*Step 4.* This step begins with the login of the user to the PLAGE platform and the subsequent upload of the BIM model. This step may be repeated throughout the design process.

*Step 5.* According to the information models implemented in PLAGE platform, the EDM server stores and manages the IFC file and sends all relevant information to the Primavera CCOP software through web services. Subsequently, Primavera CCOP lists all of the construction elements using Uniformat based on the IFC tree view.

**Figure 10:**
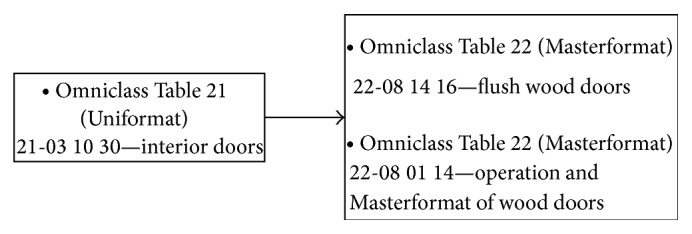
Example of link between element (interior doors) and work results.

*Step 6.* This step focused the manual link between each BIM model component and the corresponding tasks (see example in Figure 10). This action was based on the Masterformat classification (Omniclass, Table 22) and was supported by external software, the Primavera CCOP, which enables organizing and classifying construction information. The Omniclass Construction Classification System (known as Omniclass or OCCS) includes 15 tables and integrates existing systems such as Masterformat (for work results), Uniformat (for elements), and EPIC—Electronic Product Information Cooperation (for products). According to the Construction Specifications Institute [54], Omniclass was designed to classify and organize information used by the construction industry throughout project lifecycles, encompassing all types of construction. By combining tables, one can develop BIM-based project execution guides with standardized information, reducing the mapping activities and common ad hoc nature of information management [55]. 

*Step 7.* Again, using the web services implemented, the Primavera CCOP software and the EDM server communicate, and the information produced in the previous step is included in the IFC model.

*Step 8.* At this point it is important to verify the conformance of the BIM model and inherent information. The user logs into PLAGE platform and, using the BIM viewer, checks the information related to each BIM component.

*Step 9.* In this step the BIM-based interface was used to launch RFQ or RFI. By selecting the elements of BIM model, it was possible to initiate the e-procurement/consulting process. In the present case only the RFQ of general BIM components were tested, that is, windows, doors, and another building equipment. The simulation was conducted with the involvement of the client and major contractor, who participated in the research project as partners.

*Step 10.* After the client/owner triggers the RFQ, web services exports the BIM technical and contractual data from the EDM server to the Vortal eGOV to launch the RFQ process. Besides the designs and specifications, the PLAGE Platform also releases the tender documents and the templates for the bid reply of the competitors. In this process complementary information may be added, such as expected dates for execution, maximum price, and selection criteria.

*Step 11.* In order to access the RFQ information, suppliers must log into the PLAGE platform, in which they view the BIM model and consult relevant information; the BIM-IFC file may be used by suppliers to analyze and make estimates in their own software through file transfer or web services (if previously set up). 

*Step 12.* In this step suppliers used BIM viewer interface to add prices to BIM components, using a form or linking external files (such as pdf) to BIM components. The EDM server includes price information in the IFC file and creates pointers to external files that the supplier might submit. 

*Step 13.* Finally, a multicriteria evaluation tool was used, implemented on the PLAGE platform in order to support the proposal's evaluation. It can be based exclusively on price or various criteria [17].

## 7. Discussion and Major Challenges

The proposed solution of BIM e-procurement was designed with the aim of providing a richer approach to the information flows associated with procurement, as it may foster more strategic approaches to e-procurement, by improving information management potential, stimulating collaboration, and maximizing supply chain management. In traditional e-procurement platforms, collaboration arises primarily from buying requirements for procurement through the specification development process, using real-time communication and exchange of information [56, 57]. However, a BIM-based e-procurement vision may extend these capabilities to design and develop products, manufacturing processes, logistics, and distribution strategies.

The chosen case study research design consisted in a qualitative research, which allowed a description of the interaction of context and actors in a specific setting [58]. While the quantitative research is concerned with identifying relationships between variables and generalizing those results to the world at large, the qualitative research seeks to understand phenomena in depth and within specific contexts [59]. Considering this, the present research has conducted several interviews with the members of the testing team, which allowed identifying the major gains of the proposed solution.

From this specific case study, and based on the expertise of the interviewed professionals, it is possible to sustain that BIM-based e-procurement may reduce the time and effort variables related with information management activities that have heavy contractual and administrative procedures and documentation, as BIM model will serve as a unique repository to all this information, both to the owner, contractors, designers, or subcontractors. Moreover, it is expected that as the various agents involved in the procurement process may reuse BIM elements, buyers and suppliers will enhance the integrity and reliability of information used, diminishing the errors due to information operation. These benefits were clearly identified by the interviewees in qualitative research but it was not the focus of the research to measure or quantify these benefits.

However, these benefits are shadowed by the costs associated with the required additional effort to edit the documents and linkages to the BIM model and maintain a procurement process using coherent information between product model, quantities, product descriptions, and contractual arrangements centered on a BIM model. This editing requires currently specialized technical support to combine the various information sets into the BIM-based procurement process. Although the traditional process also requires editing a substantial part of the information for the procurement process, the effort does not require a unified treatment of documents and there is a more fragmented approached to information and documents management. Indeed, the approach requires a deep understanding about how to create the models and how to classify the information included in the models using predefined taxonomies and linkage of vectorial and nonvectorial information within a BIM Viewer.

Although the research project did not accurately measure the additional effort for the procurement process in comparison with traditional procurement mechanisms, the various partners involved in the process recognized that the learning curve was considerable and that it did not make the new administrative process overall more streamlined. Although the BIM-based e-procurement interface has shown itself to be useful, the success of its implementation requires a deep understanding about how to create the models and how to classify the information included in the models using predefined taxonomies. This issue is very important as in construction projects procurement of trade services and products tends to be a one-time activity, and thus there are few opportunities for gaining efficiency due to replication of the process. Moreover, there were several difficulties that emerged with the pilot case study that were not anticipated.

A fundamental hindrance was the ability to convert individual building objects in aggregate product and service “blocks” that are released to tender. The major problem is on the level of aggregation, because BIM objects tend to be very elementary and tenders focus on aggregate levels of products and services. Quantities for tendering are easy to obtain directly from the BIM model, but how to organize the elements to be tendered is a rather complex issue, and the existing models do not reflect this need. It is easy when the blocks are windows, doors, or other highly specific products but it becomes considerably harder when there is a need to aggregate with works that involve other types of products or trade services.

Some problems were identified regarding the interoperability models: when transformed into IFC the models lost some information included in the original proprietary format. It was also found that there are no specific IFC classes for procurement related information, which can hinder interoperability in procurement processes. Furthermore, it was evident that the wide implementation of the solutions proposed depends to a great extent on the dissemination of standardized taxonomies. Using predefined information classifications for BIM elements, work results, products, resources, and so forth cannot be avoided because these classifications must be common to the various actors to guarantee that the same codification will be used for a specific piece of information. This is particularly important whenever information flows across organizational boundaries, from procurer (buyer) to tenderer (supplier) that may have different modeling configurations and working practices that should be previously aligned.

Although the research project has uncovered several hurdles, the BIM-based e-procurement prototype presented in this paper demonstrated successful results. The parties involved, including the owner of the building and one of the major national general contractors, considered the solution opportune and useful and reported the importance of stimulating increasingly integrated and collaborative processes using innovative ICT. Several benefits have been identified as quite promising in terms of replicating the pilot in other full-scale construction projects. First, the need for the procurement process to input information in the BIM model has proven to be very useful as the model becomes a natural repository for all technical, managerial, administrative, and contractual information about the project. Indeed, rather than having several digital repositories of the project, agents in the project have a user-friendly interface—the BIM Viewer—for the most important documents facilitating the search, retrieval, distribution, and storage of documents and information, because they are connected to the model. This is particularly useful for all contractual procurement administrative documentation, as in construction projects these are a large part of all information. Hence, though the approach adds to the size and complexity of the BIM model, it significantly improves information management.

Second, as the buyer triggers the procurement workflow the IFC exportation of the technical and contractual data from the BIM model to the platform occurs. In addition to the architectural designs and specifications, the platform also releases the tender documents and the templates for the bid reply of the suppliers. In this process complementary information may be added, such as expected dates for execution, maximum price, selection criteria, and so forth. However, this information is incorporated in the tender documents through a structured procedure that also feeds the original BIM model. Hence, a fair amount of reusability is possible, in both directions, of the information, models, and data between agents. Of particular value is the fact that buyers and suppliers can avoid the reentry of data and re-creation of models, with all the errors and misfits that typically occur in these replication processes.

Although there was a major effort to have mainly structured information in the e-procurement process, the platform also supports some complementary unstructured information in the tender document sent by the suppliers. As a result, the BIM-IFC detailed design and the filled-in bid template may contain additional information in the form of attached files (e.g., pdf format, jpg, etc.) or possibly Web links. However, each element of unstructured information has to be linked to an object within the BIM model. This complementary information and documentation may also be incorporated directly in the BIM Model (rather than being imported along with the original file), through the manipulation of the BIM Model Viewer, thereby enriching the content of the model.

The research includes some limitations related to its complex approach (i.e., action research involving several actors, distinct perspectives, and multiple phases). Such research is demanding in terms of planning and management. In some instances the research is unable to offer complete answers to the questions raised, so the approach focused primarily on the problem setting than problem solving. Nevertheless, it achieves results reflected in various viewpoints that enrich the state of the art and leverages the pertinence of final findings.

## Appendix

The information model implemented in the EDM server in order to allow an efficient management of the project data is briefly described in the following, giving special attention to the entities implemented on the EDM server.

*(i) Project.* This entity represents a specific project, which has several phases. The “currentPhase” attribute is the phase in progress and all the historic information is stored on the “previousPhase.” Each project has a unique identifier (“instanceID”) in the server, which must be used by the client application to access project information. Each project has a dedicated workspace in the model server, where the models and other project information (“owner,” “description,” “dateCreated,” “startDate,” and “endDate”) are stored.

*(ii) ProjectPhase.* This entity represents a project phase and allows managing all the information related to that specific phase. Each phase may include a main model (“mainModel”) and other models (“models”), which may include distinct information. During a specific project phase several requests for information or quotation can be made (“RequestForQuotations”), which can then be classified into distinct stages: “OpenForQuotation,” “QuotationEvaluation,” “Execution,” and “Closed”.

*(iii) ProjectModel.* This entity represents a model in a project, which is necessarily related to a specific project phase. Several attributes were defined to characterize the model such as the model version (“version”), the model type (“modelType”), a brief description of the model (“description”), the owner of the model (“responsible”), and the creation date (“dateCreated”). An attribute to identify the status of the model (editable or blocked) was also implemented (“frozen”).

*(iv) RequestForQuotation (RFQ).* This entity allows managing the requests for information or quotation, which are always related to a specific phase. It collects the several quotations (“quotations”) and allows identifying the winning tenders (“awarded”), if applicable. Besides the information/quotations, this entity allows for attaching a model to the RFQ (“baseModel”), which becomes the support for the generation of tenders. Additionally, this entity collects generic information about the RFQ, such as the start date (“startDate”), the expected ending date (“endData”), and the RFQ status (“RFQStatus”), which can be “Open” or “Closed.”

*(v) Quotation.* This entity represents a tender/information and is related to a specific RFQ. It includes information about the RFQ process (“associatedRFQ”), the tenderer (“responsible”), submission date (“dateSubmited”), comments about tender evaluation (if applicable) (“notesOnEvaluation”), the status of the quotation (“valid”), and the IFC models related to the quotation (“models”).

On the other hand, the information model which defines the information flow based on the IFC objects included in the IFC model has three entities.

*(vi) TaskView.* This entity represents a task on the PLAGE platform, which corresponds to the ifcTask entity in the IFC standard [60]. Each task may contain several subtasks. This entity includes several attributes, such as the following:

- instanceId: identifies the task according to the ifcTask entity;
- globalId: Universally Unique Identifier (UUID), included in the correspondent ifcTask entity in the IFC file;
- name: name of the task;
- numberOfElements: states the number of IFC elements related to the task;
- state: refers to the status of the task, useful to monitor any change made to the task entity;
- propertiesChanged: indicates if any property of the task has been changed;
- plageProperties: describes the set of properties defined by the Plage platform, which are related to the correspondent “ifcTask”;
- otherProperties: any other property related to a specific “ifcTask”;
- elementsChanged: indicates if any element related to the task has been changed;
- elements: defines the set of elements related to the task;
- tasksChanged: indicates if the task has been changed;
- numberOfChilds: states the number of subtasks of the task;
- childTasks: defines the subtasks of the task;
- isLastVersion: states if the entity is the last version of the task.

*(vii) ElementView.* This entity represents an IFC object and all the elements related to the IFC standard entity “ifcElement” [61]. This entity includes several attributes, such as the following:

- instanceId: identifies an element according to the ifcElement entity;
- globalId: Universally Unique Identifier (UUID), included on the correspondent “ifcElement” entity on the IFC file;
- name: name of the element;
- description: description of the IFC element;
- propertiesChanged: indicates if the properties of the element have been changed;
- plageProperties: describes the set of properties defined by the Plage platform, which are related to the correspondent “ifcElement”;
- otherProperties: any other property related to the correspondent “ifcElement”;
- elementType: refers the type of IFC element;
- state: indicates the status of the element allowing for the monitor ing of any change in task structure; concretely it tells if the element has been included or removed from a specific task;
- isLastVersion: states if the entity is the last version of the element;
- parentSpatialStructureName: indicates the name of the spatial structure in which the IFC element is inserted;
- parentSpatialStructureID: shows the identifier of the spatial structure in which the IFC element is inserted;
- parentSpatialStructureType: type of the spatial structure in which the IFC element is inserted.

*(viii) PropertyView.* This entity represents a property related to the IFC element and corresponds to the IFC entity “ifcProperty.” This entity includes several attributes, such as the following:

- instanceId: identifies the property according to the ifcProperty entity;
- name: name of the property;
- description: description of the IFC property;
- units: SI unit of the property;
- propertyType: type of property;
- propertySetName: name of the property set to which the property belongs;
- changed: indicates if the property has been changed;
- propertyValue: defines the value corresponding to the property [62].

Several web services have been also implemented in the EDM server to satisfy the management requirements of the PLAGE platform, such as the following:

- access control services: login and logout;
- project management services: CreateProject, addModelToCurrentProjectPhase, getProjectById, and getProjectByWorkspace;
- model management services: uploadProjectModel, DownloadProjectModel, getTasksAndQuantities, getTaskElements, updateTasksAndQuantities, getAllRootTasks, getAllModelElements, and getModelElementsOfType.
